# Association Between Material Hardship in Families With Young Children and Federal Relief Program Participation by Race and Ethnicity and Maternal Nativity

**DOI:** 10.1001/jamahealthforum.2023.0508

**Published:** 2023-04-21

**Authors:** Félice Lê-Scherban, Stephanie Ettinger de Cuba, Allison Bovell-Ammon, Sharon Coleman, Lindsey Rateau, Diana Cutts, Maureen Black, Timothy Heeren, Deborah A. Frank

**Affiliations:** 1 Dornsife School of Public Health, Drexel University, Philadelphia, Pennsylvania; 2 Boston University School of Public Health, Boston, Massachusetts; 3 Department of Pediatrics, Boston University Chobanian & Avedisian School of Medicine, Boston, Massachusetts; 4 Department of Pediatrics, Boston Medical Center, Boston, Massachusetts; 5 Biostatistics and Epidemiology Data Analytics Center, Boston University School of Public Health, Boston, Massachusetts; 6 Department of Pediatrics, Hennepin County Medical Center, Minneapolis, Minnesota; 7 Department of Pediatrics, University of Maryland School of Medicine, Baltimore, Maryland; 8 RTI International, Research Triangle Park, North Carolina; 9 Department of Biostatistics, Boston University School of Public Health

## Abstract

**Importance:**

Even brief periods of hardship during early childhood may have lifelong consequences. Prior cross-sectional research limited to respondents with English proficiency and internet access during the COVID-19 crisis documented families with young children that struggled to afford basic needs like food and rent. Few studies have examined experiences of families with young children by race and ethnicity and maternal nativity.

**Objective:**

To examine the association of COVID-19 relief programs with the mitigation of household food insecurity among families with young children, as well as being behind on rent and disparities in program receipt.

**Design, Setting, and Participants:**

This cohort study used data from the ongoing repeat cross-sectional Children’s HealthWatch study comprising families surveyed at baseline (January 1, 2018, to March 20, 2020) and during the COVID-19 crisis (September 1, 2020, to June 30, 2021). Baseline questionnaires were administered face to face to caregivers of children aged 48 months or younger in English or Spanish in emergency departments or primary care clinics in 5 US cities. The follow-up questionnaires were administered via telephone.

**Exposures:**

Supplemental Nutrition Assistance Program (SNAP) participation or receipt of at least 1 Economic Impact Payment (EIP; ie, stimulus check) during the COVID-19 crisis.

**Main Outcomes and Measures:**

The primary outcomes were household food insecurity (assessed via the US Household Food Security Survey Module: Six-Item Short Form) and being behind on rent. Logistic and binomial regression models were used to calculate adjusted odds ratios (aORs) and unadjusted and adjusted prevalence ratios (PRs).

**Results:**

Of 1396 caregiver-child dyads (20.3% response rate), race and ethnicity data were available for 1357 caregivers: 514 (37.9%) were Black, non-Latino; 558 (41.1%) were Latino; 230 (16.9%) were White, non-Latino; and 55 (4.1%) were of other non-Latino race or ethnicity. Among 1390 responses with nonmissing data, 417 children (30.0%) had an immigrant mother, and among 1388 responses, 1238 (33.5%) were publicly insured. During the crisis, 467 of 1395 respondents (33.5%) reported household food insecurity, and 567 of 1391 respondents (40.8%) reported being behind on rent. Families with immigrant mothers had lower odds of EIP and SNAP participation than families with US-born mothers (eg, aOR, 0.07 [95% CI, 0.05-0.12] for both EIP and SNAP vs neither), despite being more likely to report household food insecurity (adjusted PR [aPR], 1.48 [95% CI, 1.28-1.71]) and being behind on rent (aPR, 1.14 [95% CI, 1.00-1.30]). Families with Black (unadjusted PR [uPR], 1.40 [95% CI, 1.08-1.82]) or Latino (uPR, 1.54 [95% CI, 1.19-1.98]) caregivers or caregivers of other race and ethnicity (uPR, 1.67 [1.12-2.49]) were also more likely than families with White, non-Latino caregivers to experience household food insecurity or being behind on rent (families with Black caregivers: uPR, 2.02 [95% CI, 1.58-2.58]; families with Latino caregivers: 1.68 [95% CI, 1.30-2.16]; families with caregivers of other race or ethnicity: uPR, 1.94 [95% CI, 1.34-2.80]). Adjustment for covariates and differential participation in relief programs did not entirely account for these disparities.

**Conclusions and Relevance:**

The results of this cohort study suggest that the COVID-19 crisis exacerbated preexisting inequities in food insecurity and difficulty paying rent according to race and ethnicity and maternal nativity and that equity-focused policy changes are needed to ensure that all children and their families in the US can afford basic needs for optimal health.

## Key Points

**Question:**

What was the association between participation in federal COVID-19 relief programs and mitigation of food and housing hardships and disparities among families with young children?

**Findings:**

In this cohort study using interviews before and during the COVID-19 crisis of 1396 caregivers with children 48 months or younger, receipt of benefits from the Supplemental Nutrition Assistance Program and/or Economic Impact Payments was associated with reduced prevalence of household food insecurity and being behind on rent. However, there were disparities in receipt of benefits as well as food insecurity and being behind on rent by maternal nativity and caregiver race and ethnicity.

**Meaning:**

The findings of this study suggest that equity-driven policy design is needed to ensure that benefits reach all families, especially among historically marginalized groups.

## Teaser

This cohort study of data from questionnaires administered before and during the COVID-19 crises to caregivers of children aged 48 months or younger examines the association of participation in COVID-19 relief programs with the mitigation of household food insecurity and being behind on rent as well as disparities in program participation among families with young children.

## Introduction

Economic hardships—including inability to afford food or rent—are strongly associated with adverse health outcomes across the life span.^1,2,3^ Food insecurity, defined as inadequate access to enough food for all household members to live active, healthy lives, is associated with poor physical and mental health among children and adults, developmental delays in children, increased hospitalizations, and avoidable health care costs.^1,4,5,6^ Inability to pay rent on time places families at risk of eviction and is associated with poor physical health among children and adults, maternal depression, and child hospitalizations.^3^

Young children are particularly vulnerable to adverse outcomes associated with hardship.^3,7,8^ The rapidly growing brain of an infant or toddler requires consistent high-quality nutrition and stable housing for healthy development.^9^ Even brief periods of hardship and stress during early childhood may have lifelong consequences.^8,10^

The onset of the COVID-19 pandemic initiated a period of widespread job loss and disruption due to business, school, and child care closures necessary to slow the rapid spread of the virus. Research conducted early in the pandemic documented that families with young children reported struggling to meet basic needs like food and rent due to pandemic-related closures and economic distress.^11,12^ National surveys found that families with children, Black and Latino adults, and immigrants consistently reported higher rates of economic hardship before the pandemic^13^ and disproportionately experienced hardships during the pandemic compared with the population overall. These inequities reflect persistent structural racism and xenophobia.^14,15^

To mitigate the effects of the COVID-19 crisis, the US Congress passed a series of relief packages between March 11, 2020, and March 25, 2021, allocating trillions of dollars in aid to bolster the US economy and alleviate economic hardships for families and individuals. Federal relief efforts, including investment in nutrition assistance and direct cash transfers, buffered US households from hardships during the crisis.^16^ Among the largest investments to facilitate households with low and moderate incomes meeting basic needs were streamlined access to and boosted benefit levels of the Supplemental Nutrition Assistance Program (SNAP), the largest antihunger program in the US, and rapid deployment of Economic Impact Payments (EIPs), also known as stimulus checks. During the early pandemic, SNAP enrollment increased from 36 million people in 2019 to 44 million in 2020 as the program expanded, as designed, to meet the needs of people experiencing food insecurity.^17^ The Internal Revenue Service disbursed 3 EIPs to over 92 percent of households over the course of 1 year in amounts ranging from $600 to $1400 per adult and $500 to $1400 per child.^18,19^ While there were no restrictions on the use of payments, families, especially those with lower incomes, used payments to meet basic needs, potentially alleviating economic hardships.^20^

Multiple relief policies, including SNAP and distribution of EIPs, excluded or limited access to immigrants, imposed administrative barriers to participation for families with low incomes, and were inadequate for families with the greatest needs. These practices disproportionately affected families with Black, Latino, or immigrant members.^21^ Anti-immigrant rhetoric, exclusionary immigration policies, and changing eligibility rules for immigrants in relief programs resulted in millions of families with immigrant members not receiving public assistance that could have mitigated hardships.^22,23,24^

Few existing studies specifically focus on experiences of families with young children by race and ethnicity and maternal nativity.^25^ The current study uses prospective data to examine the association of federal relief programs with mitigation of food insecurity and being behind on rent and illuminates disparities in program participation. We hypothesized (1) that there would be disparities in SNAP and EIP participation with lower participation among families with caregivers who were Black or Latino or that had an immigrant mother, (2) that SNAP and EIP participation would be associated with less food insecurity and being behind on rent compared with nonparticipation, and (3) that, in exploratory analysis, disparities in SNAP and EIP participation would partially account for disparities in food insecurity and being behind on rent during the COVID-19 crisis.

## Methods

### Study Population

Data come from the ongoing repeat cross-sectional Children’s HealthWatch (CHW) study, conducted since June 23, 1998,^26^ and the CHW Follow-up Study (CHW-COVID), a longitudinal sentinel cohort study of families surveyed in English or Spanish before and during the pandemic. Baseline questionnaires were administered face to face between January 1, 2018, and March 20, 2020 (baseline) in an emergency department or primary care clinic in 5 cities: Boston, Massachusetts; Baltimore, Maryland (with 2 sites, 1 of each type); Minneapolis, Minnesota; Philadelphia, Pennsylvania; and Little Rock, Arkansas.^27^ Each research site received institutional review board approval annually. Eligibility criteria at all sites included state residency, child age of 48 months or younger, English- or Spanish-speaking primary caregiver, caregiver residence in the child’s household, and consent to be interviewed (written or verbal, depending on the site). Questionnaires^26,28^ were administered by trained interviewers and included sociodemographic characteristics, household material hardships, and public benefit program participation. The telephone CHW-COVID follow-up questionnaires (administered between September 1, 2020, and June 30, 2021) included questions about material hardships and program participation, including SNAP and EIP, since the start of the crisis.^28^ There were 2 versions of the follow-up questionnaire. If the same caregiver in the household was interviewed, the first version and the baseline sociodemographic data were used. If a different caregiver in the household (eg, the father vs the mother) was interviewed, the second version, which included that individual’s sociodemographic information (eg, race and ethnicity), was used. The study followed the Strengthening the Reporting of Observational Studies in Epidemiology (STROBE) reporting guideline for cohort studies.

### Measures

In both baseline and follow-up questionnaires, caregivers reported on household food insecurity,^29^ using the validated US Household Food Security Survey Module: Six-Item Short Form,^30^ and on whether they were behind on rent or mortgage (hereinafter behind on rent), a risk factor for housing instability, including eviction. For household food insecurity, 0 or 1 question affirmed was coded as food secure and 2 or more questions affirmed was coded as food insecure. For both food insecurity and being behind on rent, the reference period at baseline was within the past 12 months and at follow-up was since the start of the COVID-19 pandemic. Both hardship outcomes were scored dichotomously and analyzed separately.

We created a 4-category measure of program participation during the COVID-19 pandemic: neither SNAP nor EIP, SNAP only, EIP only, and both SNAP and EIP. Caregiver race and ethnicity were based on their answers and categorized as Black, non-Latino; Latino; White, non-Latino; or other or multiple non-Latino race or ethnicity (hereinafter other race or ethnicity), which included American Indian or Native American and Asian or Pacific Islander participants, although the numbers of participants in this category were too small to analyze independently. Data on race and ethnicity were collected because it is important to understand how the COVID-19 crisis and related public policy and associations with family health and hardship varied by race and ethnicity. Maternal nativity was categorized as US born (including US territories) or immigrant. Covariates, chosen a priori, were caregiver education, number of children in the household, child age, children’s health insurance type (as a proxy for household income), and whether anyone in the household was employed.

### Statistical Analysis

We tested our first hypothesis, that there would be disparities in SNAP and EIP participation, with covariate-adjusted multinomial logistic regression analysis. We used the category neither SNAP nor EIP as the reference outcome group and included either race and ethnicity or maternal nativity as the predictors of interest. Results are presented as adjusted odds ratios (aORs) with 95% CIs. The independence of irrelevant alternatives assumption of multinomial logistic regression requires that there be no other alternative outcome choices that, if present, would change the odds of the outcome choices examined. For our analysis of SNAP and EIP participation, there were no competing benefit programs that would replace the value of SNAP or EIP.

We tested our second hypothesis, that SNAP and EIP participation would be associated with less food insecurity and being behind on rent, using adjusted log-binomial regression models with household food insecurity and being behind on rent as outcomes and program participation as the predictor of interest. Results are presented as adjusted prevalence ratios (aPRs) with 95% CIs. Finally, we tested our third hypothesis, that disparities in hardships might be partially attributable to disparities in SNAP and EIP participation, using log-binomial regression models with household food insecurity and being behind on rent as outcomes. Results are presented as prevalence ratios (PRs) with 95% CIs. We used an exploratory mediation analysis approach in which we compared race and ethnicity– and nativity-based disparities in the outcomes before and after adjustment for program participation; attenuation of the disparities after adjustment for program participation would support our hypothesis. We assessed statistical significance using 2-tailed tests with α = .05; 2-sided *P* values represent results from comparing each characteristic by level of program participation. Each test was performed using χ^2^ analysis for categorical characteristics and analysis of variance for independent samples. Data were analyzed using SAS, version 9.4 (SAS Institute Inc).

## Results

Out of 6875 eligible caregivers interviewed in person before the pandemic and recontacted, 1396 participated in the telephone follow-up (20.3% response rate). The mean (SD) time between baseline and follow-up questionnaire was 24.0 (7.8) months. The follow-up cohort did not differ from the prepandemic baseline cohort in household income, marital status, SNAP participation at baseline, or caregiver or household employment. The proportion of Latino and immigrant caregivers in the follow-up cohort was higher than at baseline. Among the 1396 caregiver-child dyads, race and ethnicity data were available for 1357 caregivers; 514 (37.9%) caregivers were Black, non-Latino; 558 (41.1%) were Latino; 230 (16.9%) were White, non-Latino; and 55 (4.1%) were of other race or ethnicity. Among 1390 responses with nonmissing data, 417 (30.0%) children had an immigrant mother (Table 1). Among 1344 responses, 1086 caregivers (80.8%) were renters; among 1393 responses, 716 caregivers (51.4%) had no education beyond high school, and nearly all children (1238 of 1388 responses; 89.2%) were publicly insured. The mean (SD) child age was 47.0 (15.4) months.

**Table 1.** Sociodemographic Characteristics of the Study Participants According to Participation in Relief Programs

Characteristic^a^	Participants, No. (%)					*P* value^b^
	All	No program participation	SNAP only	EIP only	SNAP and EIP	
Total participants, No. (%)	1396 (100.0)	148 (10.6)	161 (11.5)	529 (37.9)	558 (40.0)	NA
Caregiver race and ethnicity (n = 1357)						
Black, non-Latino	514 (37.9)	23 (16.7)	49 (31.2)	195 (37.9)	247 (45.1)	<.001
Latino	558 (41.1)	96 (69.6)	96 (61.1)	142 (27.6)	224 (40.9)	
White, non-Latino	230 (16.9)	13 (9.4)	7 (4.5)	156 (30.4)	54 (9.9)	
Other race or ethnicity^c^	55 (4.1)	6 (4.3)	5 (3.2)	21 (4.1)	23 (4.2)	
Maternal nativity (n = 1390)						
US born	973 (70.0)	38 (25.7)	83 (51.6)	394 (75.0)	458 (82.4)	<.001
Immigrant	417 (30.0)	110 (74.3)	78 (48.4)	131 (25.0)	98 (17.6)	
Study site						
Baltimore, MD	131 (9.4)	5 (3.4)	16 (9.9)	38 (7.2)	72 (12.9)	<.001
Boston, MA	297 (21.3)	29 (19.6)	45 (28.0)	106 (20.0)	117 (21.0)	
Little Rock, AR	388 (27.8)	37 (25.0)	11 (6.8)	220 (41.6)	120 (21.5)	
Minneapolis, MN	172 (12.3)	51 (34.5)	23 (14.3)	65 (12.3)	33 (5.9)	
Philadelphia, PA	408 (29.2)	26 (17.6)	66 (41.0)	100 (18.9)	216 (38.7)	
Child age at follow-up, mean (SD), mo	47.0 (15.4)	45.1 (15.4)	44.2 (16.0)	48.8 (15.3)	46.5 (15.2)	<.001
Caregiver education (n = 1393)						
<High school diploma	216 (15.5)	49 (33.3)	49 (30.6)	39 (7.4)	79 (14.2)	<.001
High school diploma or GED	500 (35.9)	55 (37.4)	61 (38.1)	160 (30.3)	224 (40.1)	
Technical or postsecondary degree	677 (48.6)	43 (29.3)	50 (31.3)	329 (62.3)	255 (45.7)	
Child insurance (n = 1388)						
Public	1238 (89.2)	126 (85.7)	152 (95.6)	420 (79.8)	540 (97.1)	<.001
Private	130 (9.4)	15 (10.2)	1 (0.6)	101 (19.2)	13 (2.3)	
No insurance	20 (1.4)	6 (4.1)	6 (3.8)	5 (1.0)	3 (0.5)	
Household employment (n = 1378)						
Yes	805 (58.4)	65 (44.8)	49 (31.2)	377 (71.7)	314 (57.1)	<.001
No	573 (41.6)	80 (55.2)	108 (68.8)	149 (28.3)	236 (42.9)	
No. of children in household, mean (SD)	2.6 (1.3)	2.3 (1.2)	2.7 (1.3)	2.6 (1.3)	2.7 (1.4)	.01
Months since baseline questionnaires, mean (SD)	24.0 (7.8)	21.9 (7.9)	22.6 (6.8)	24.7 (8.1)	24.2 (7.7)	<.001
Renter household (n = 1344)	1086 (80.8)	114 (78.6)	139 (92.1)	156 (69.8)	473 (88.9)	<.001
Household food insecurity at baseline (n = 1395)						.
Yes	292 (20.9)	21 (14.2)	36 (22.5)	99 (18.7)	136 (24.4)	.02
No	1103 (79.1)	127 (85.8)	124 (77.5)	430 (81.3)	422 (75.6)	
Household food insecurity during COVID-19 crisis						
Yes	467 (33.5)	66 (44.6)	67 (41.9)	154 (29.1)	180 (32.3)	<.001
No	929 (66.5)	82 (55.4)	94 (58.1)	375 (70.9)	378 (67.7)	
Behind on rent/mortgage at baseline (n = 1347)						
Yes	241 (17.9)	19 (12.9)	22 (14.4)	73 (14.1)	127 (23.9)	<.001
No	1106 (82.1)	128 (87.1)	131 (85.6)	443 (85.9)	404 (76.1)	
Behind on rent/mortgage during COVID-19 crisis (n = 1391)						
Yes	567 (40.8)	72 (48.6)	67 (41.9)	173 (32.7)	255 (46.0)	<.001
No	824 (59.2)	76 (51.4)	93 (58.1)	356 (67.3)	299 (54.0)	

Abbreviations: EIP, Economic Impact Payment; GED, General Educational Development; NA, not applicable; SNAP, Supplemental Nutrition Assistance Program.

^a^ Denominators in some groups differ because of missing data; percentages in columns therefore reflect the differing denominators in the subcategories.

^b^ *P* values represent results from comparing each characteristic by level of program participation. Each test was performed using χ^2^ analysis for categorical characteristics and analysis of variance for independent samples.

^c^ Other races and ethnicities included American Indian or Native American and Asian or Pacific Islander.

Over one-third of caregivers (467; 33.5%) reported household food insecurity during the COVID-19 crisis compared with 292 (20.9%) at baseline (Table 1). Being behind on rent also increased dramatically during the crisis, more than doubling, from 241 (17.9%) at baseline to 567 (40.8%). Regarding participation in relief programs, 148 caregivers (10.6%) participated in neither SNAP nor EIP, 161 (11.5%) participated in SNAP only, 529 (37.9%) participated in EIP only, and 558 caregivers (40.0%) reported participating in both SNAP and EIP during the COVID-19 crisis.

As hypothesized, compared with participation in neither program, families with Latino caregivers and caregivers of other race or ethnicity had lower odds of EIP participation than families with White, non-Latino caregivers (aOR, 0.16 [95% CI, 0.08-0.31] and aOR, 0.26 [95% CI, 0.09-0.77], respectively) (Table 2). Odds of participation in both programs or in SNAP only were not statistically significantly different from those among families with White, non-Latino caregivers. Counter to our hypothesis, families with Black, non-Latino caregivers had higher but nonsignificant odds of participation in SNAP only (aOR, 2.71 [95% CI, 0.91-8.03]) and both programs (aOR, 1.93 [95% CI, 0.87-4.28]) compared with families with White non-Latino caregivers. Consistent with our hypothesis, families with immigrant mothers had far lower odds of program participation than families with US-born mothers (aOR, 0.30 [0.18-0.49] for SNAP only; aOR, 0.15 [0.10-0.23] for EIP only; aOR, 0.07 [0.05-0.12] for both programs).

**Table 2.** Adjusted Odds Ratios of COVID-19 Relief Program Participation by Caregiver Race and Ethnicity and Maternal Nativity

Characteristic	aOR (95% CI)^a^		
	SNAP only (vs neither)	EIP only (vs neither)	Both SNAP and EIP (vs neither)
Race and ethnicity^b^^,^^c^			
Black, non-Latino	2.71 (0.91-8.03)	0.74 (0.34-1.59)	1.93 (0.87-4.28)
Latino	1.03 (0.37-2.83)	0.16 (0.08-0.31)	0.40 (0.20-0.83)
White, non-Latino	1 [Reference]	1 [Reference]	1 [Reference]
Other race or ethnicity	0.72 (0.13-3.91)	0.26 (0.09-0.77)	0.76 (0.25-2.34)
Maternal nativity^d^			
US born	1 [Reference]	1 [Reference]	1 [Reference]
Immigrant	0.30 (0.18-0.49)	0.15 (0.10-0.23)	0.07 (0.05-0.12)

Abbreviations: aOR, adjusted odds ratio; EIP, Economic Impact Payment; SNAP, Supplemental Nutrition Assistance Program.

^a^ From multinomial logistic regression models adjusted for caregiver education, number of children in the household, child age, child health insurance, and any employment in the household.

^b^ Other race and ethnicity was defined as other or multiple non-Latino races or ethnicities and included American Indian or Native American and Asian or Pacific Islander participants.

^c^ For complete-case analysis, n = 1332 for model of race and ethnicity.

^d^ For complete-case analysis, n = 1365 for model of maternal nativity.

Table 3 shows adjusted PRs (aPRs) for relief program participation with household food insecurity and being behind on rent during the COVID-19 crisis. All point estimates were in the hypothesized direction, although not all results were statistically significant. Compared with participation in neither program, participation in SNAP only was not associated with lower prevalence of household food insecurity (adjusted PR [aPR], 0.84 [95% CI, 0.65, 1.08]), while participation in EIP only or both SNAP and EIP was associated with approximately 25% lower prevalence of household food insecurity (EIP only: aPR, 0.73 [95% CI, 0.59-0.91]; both: aPR, 0.76 [95% CI, 0.60-0.96]). Compared with families participating in neither program, those participating in SNAP were more than 20% less likely to report being behind on rent (aPR, 0.78 [95% CI, 0.61-0.99]), and those participating in EIP only were approximately 30% less likely to report being behind on rent (aPR, 0.71 [95% CI, 0.58-0.86]). Although the prevalence of being behind on rent was lower with participation in both programs, the result was not statistically significant (aPR, 0.86 [95% CI, 0.72-1.03]).

**Table 3.** Adjusted Prevalence Ratios of Household Food Insecurity and Being Behind on Rent by Relief Program Participation

Relief program	aPR (95% CI)^a^	
	Household food insecurity^b^	Behind on rent^c^
Neither SNAP nor EIP	1 [Reference]	1 [Reference]
SNAP only	0.84 (0.65-1.08)	0.78 (0.61-0.99)
EIP only	0.73 (0.59-0.91)	0.71 (0.58-0.86)
SNAP and EIP	0.76 (0.60-0.96)	0.86 (0.72-1.03)

Abbreviations: aPR, adjusted prevalence ratio; EIP, Economic Impact Payment; SNAP, Supplemental Nutrition Assistance Program.

^a^ From log-binomial regression models adjusted for caregiver education, number of children in the household, child age, child health insurance, and household employment.

^b^ For complete-case analysis, n = 1371 for model of food insecurity.

^c^ For complete-case analysis, n = 1368 for model of being behind on rent.

Table 4 shows results from exploratory analyses to test our hypothesis that relief program participation mediated race and ethnicity– and nativity-based disparities in household food insecurity and being behind on rent during the COVID-19 crisis. There were disparities in both outcomes by race and ethnicity and nativity. Compared with families with White, Non-Latino caregivers, families with Black, non-Latino caregivers (unadjusted PR [uPR], 1.40 [95% CI, 1.08-1.82]), Latino caregivers (uPR, 1.54 [95% CI, 1.19-1.98]), and caregivers of other race or ethnicity (uPR, 1.67 [95% CI, 1.12-2.49]) were more likely to report household food insecurity (Table 4, model 1). Compared with families with US-born mothers, families with immigrant mothers were also more likely to report household food insecurity (uPR, 1.63 [95% CI, 1.41-1.88]). Compared with families with White, non-Latino caregivers, families of other racial and/or ethnic groups were nearly more likely to report being behind on rent (families with Black, non-Latino caregivers: uPR, 2.02 [95% CI, 1.58-2.58]; families with Latino caregivers: uPR, 1.68 [95% CI, 1.30-2.16]; and families with caregivers of other race and ethnicity: uPR, 1.94 [95% CI, 1.34-2.80]). Families with immigrant mothers were also more likely to report being behind on rent compared with families with US-born mothers (uPR, 1.21 [95% CI, 1.06-1.37]) (Table 4, Model 1). Adjustment for covariates only moderately attenuated these disparities (Table 4, Model 2), and additional adjustment for SNAP and EIP participation somewhat attenuated these disparities for some groups and outcomes (Table 4, Model 3).

**Table 4.** Association of COVID-19 Relief Program Participation With Race and Ethnicity– and Nativity-Based Disparities in Household Food Insecurity and Being Behind on Rent

Characteristic	PR (95% CI)^a^					
	Household food insecurity^b^			Behind on rent^c^		
	Model 1: unadjusted	Model 2: covariate adjusted	Model 3: covariate adjusted with relief program participation	Model 1: unadjusted	Model 2: covariate adjusted	Model 3: covariate adjusted with relief program participation
Caregiver race and ethnicity						
Black, non-Latino	1.40 (1.08-1.82)	1.23 (0.94-1.60)	1.18 (0.92-1.52)	2.02 (1.58-2.58)	1.75 (1.35-2.26)	1.70 (1.31-2.20)
Latino	1.54 (1.19-1.98)	1.21 (0.93-1.57)	1.13 (0.88-1.45)	1.68 (1.30-2.16)	1.36 (1.05-1.78)	1.28 (0.98-1.68)
White, non-Latino	1 [Reference]	1 [Reference]	1 [Reference]	1 [Reference]	1 [Reference]	1 [Reference]
Other race or ethnicity^d^	1.67 (1.12-2.49)	1.49 (1.00-2.23)	1.36 (0.92-2.01)	1.94 (1.34-2.80)	1.83 (1.28-2.62)	1.71 (1.19-2.45)
Maternal nativity						
US born	1 [Reference]	1 [Reference]	1 [Reference]	1 [Reference]	1 [Reference]	1 [Reference]
Immigrant	1.63 (1.41-1.88)	1.48 (1.28-1.71)	1.41 (1.21-1.65)	1.21 (1.06-1.37)	1.14 (1.00-1.30)	1.11 (0.97-1.29)

Abbreviation: PR, prevalence ratio.

^a^ From log-binomial regression models: models 2 and 3 adjusted for caregiver education, number of children in the household, child age, child health insurance, and household employment. Model 3 additionally adjusted for participation in the Supplemental Nutrition Assistance Program and receipt of an Economic Impact Payment.

^b^ For complete-case analysis, n = 1332 for model of food insecurity by race and ethnicity and n = 1365 for model by maternal nativity.

^c^ For complete-case analysis, n = 1329 for model of being behind on rent by race and ethnicity and n = 1362 for model by maternal nativity.

^d^ Other race and ethnicity was defined as other or multiple non-Latino races or ethnicities and included American Indian or Native American and Asian or Pacific Islander participants.

## Discussion

This cohort study yields timely, policy-relevant information for advancing health equity among families with young children, an understudied population. We found that families with immigrant mothers had lower odds of COVID-19–related relief program participation than families with US-born mothers, although families with immigrant mothers, as well as those with Black or Latino caregivers (compared with White, non-Latino mothers), were more likely to experience household food insecurity or being behind on rent, hardships relief programs were designed to address. In this sample, differential participation in the relief programs examined did not fully account for the disparities in household hardships.

In the US, one-quarter of children live with at least 1 immigrant parent and more than 40% of children are Black or Latino.^31,32^ Well-documented disparities in hardships across these groups, in comparison with White, non-Latino children with US-born parents, likely contribute to health inequities in food insecurity and housing instability.^2,33^ A sentinel cohort study among families with young children also documented dramatic increases in economic hardships during the pandemic, but did not disaggregate by race, ethnicity, or nativity.^12^ Our results are consistent with previous studies^11,21,22,23,24,34^ documenting disproportionate associations of the COVID-19 crisis with health and economic hardship for Black, Latino, and immigrant populations.

After disbursement of EIPs, research^35^ using US Census data found reductions in the prevalence of food insufficiency, a construct related to food insecurity, and being behind on rent, particularly among families with children and households with incomes under $25 000. Data also suggest SNAP participation reduced food insecurity risk among adults experiencing financial challenges early in the crisis.^36^ While our findings showed that relief program participation attenuated associations of race and ethnicity and nativity with food and housing hardships, results did not fully support the hypothesis that differential participation explained disparities in household hardships in our sample. Our results also showed higher, though nonsignificant, participation rates among Black families compared with White and Latino families in SNAP and SNAP and EIP, potentially due to better outreach to connect families to food resources and, in particular, COVID-19–related removal of barriers to enrollment in SNAP.

Our results illuminate the persistence of substantial hardship disparities among families with young children even in the presence of federal COVID-19 relief programs. This represents a missed opportunity for policies that not only alleviate hardships among all families but also reduce disparities. Economic Impact Payment policy design and implementation may have blunted the payments’ effect on reducing racial and national origin disparities. Specifically, eligibility criteria for EIP participation created under the Coronavirus Aid, Relief, and Economic Security (CARES) Act, passed in March 2020, barred from eligibility families with members without Social Security numbers. This meant that nearly 3 million US citizens and lawfully present immigrants in mixed immigration–status families were excluded from payments.^37^ While the second round of EIPs, passed in December 2020, extended eligibility to some mixed immigration–status families, exclusions persisted.^38^ The disbursement model for EIPs may have also reduced the policy’s ability to address disparities. Families who filed taxes in the previous 2 years were automatically assessed for eligibility and, if eligible, sent payments, but this structure resulted in substantial barriers for over 12 million low-income households^39^ that were not required to file taxes during that period.

The policy design of the SNAP benefits increase included in the CARES Act may also have contributed to failure to reduce disparities. Families that receive the maximum benefit have the lowest incomes, as the SNAP calculation deems that they have no other income for household food purchases. However, the initial structure of the policy change in response to the COVID-19 crisis increased benefits to the maximum amount for all participants. That meant those already receiving the maximum benefit amount did not initially receive an increase. This was rectified with passage of subsequent relief packages. Nevertheless, preexisting SNAP eligibility requirements also meant that otherwise eligible lawfully present immigrants who had arrived in the US within the previous 5 years remained ineligible for the program.^40,41,42^

In addition to exclusions and barriers in relief policies, the disparities observed in this study among families with immigrant mothers may also have been associated with well-documented,^40,42,43,44,45^ chilling effects of increasingly exclusionary immigration policies and harmful rhetoric around immigrant access to public benefits. Recent research^40,42,43,44,45^ shows lawfully present immigrants eligible for assistance remained fearful of accessing benefits for which they or their children were eligible.

This study may prove instructive for ongoing policy conversations related to another cash transfer program administered through the US tax system, the advance Child Tax Credit (CTC). The advance CTC was more widely available to families with children than EIPs and was associated with positive outcomes.^46,47,48^ Legislation to expand the advance the CTC and remove immigration exclusions and reduce barriers to access may provide a transformative opportunity to reduce disparities and advance equity among families with young children.

Further research is necessary to understand how subsequent changes in policy design, including increases in SNAP benefits and the implementation of a third and more inclusively distributed EIP, acting together with other COVID-19–era policies like the disbursement of advance CTC payments, may impact disparities in household hardships among families with young children. The rapidly changing policy landscape and the sunsetting of many relief policies may deepen disparities in economic hardships by race, ethnicity, and nativity over time.

### Strengths and Limitations

This study has several strengths, including its multisite, prospective design with wide diversity of participant race, ethnicity, and nativity. Moreover, the study provides data on an underrepresented group of families with young children that is both difficult to reach through online questionnaires, given generally unreliable internet access, and highly vulnerable to economic hardship.^49^

This study also has limitations. The sample is not nationally representative, although it is drawn from a sentinel study design intended to monitor a population at highest risk of showing early signs of either benefit or harm^9^ from a given exposure. The sample is similar in family demographic characteristics and child health outcomes to low-income families in the National Survey of Children’s Health.^50^ All hardships, program participation, and other data were self-reported and thus subject to recall bias, although the food security measure is validated^30^ and the measure of being behind on rent has strong criterion validity and has been used in several studies.^3,51^ The other race or ethnicity category had a small sample size and should be interpreted with caution. We were not able to further disaggregate racial and ethnic groups because of small sample sizes, which also resulted in wide CIs for some outcomes.

## Conclusions

This cohort study found significant race and ethnicity– and nativity-based disparities in food and housing-related hardships among families with young children during the COVID-19 crisis, coupled with significant differences in participation in relief programs to address these hardships. Inclusive, equity-driven policy design is as important as rapid deployment of direct cash transfer payments to ensure that benefits reach all families, especially historically marginalized groups. Specifically, legislators and other leaders must enact and equitably implement robust policies that reduce disparities by removing immigration-related exclusions and eliminating access barriers for families with very low incomes and Black and Latino families. The pandemic brought great economic and societal change, exacerbating preexisting inequities. Thus, the times require equally great policy change to not just reduce but eradicate inequities in the US to ensure that all families with children can afford the food and housing they need to be healthy.

## Notes

Supplement 1.
Data Sharing Statement

## References

[1] Gundersen C, Ziliak JP. Food insecurity and health outcomes. Health Aff (Millwood). 2015;34(11):1830-1839. doi:10.1377/hlthaff.2015.0645 26526240

[2] Hernández D, Jiang Y, Carrión D, Phillips D, Aratani Y. Housing hardship and energy insecurity among native-born and immigrant low-income families with children in the United States. J Child Poverty. 2016;22(2):77-92. doi:10.1080/10796126.2016.1148672 27616875 PMC5016025

[3] Sandel M, Sheward R, Ettinger de Cuba S, . Unstable housing and caregiver and child health in renter families. Pediatrics. 2018;141(2):e20172199. doi:10.1542/peds.2017-2199 29358482

[4] Shankar P, Chung R, Frank DA. Association of food Insecurity with children’s behavioral, emotional, and academic outcomes: a systematic review. J Dev Behav Pediatr. 2017;38(2):135-150. doi:10.1097/DBP.0000000000000383 28134627

[5] Cook JT, Frank DA, Berkowitz C, . Food insecurity is associated with adverse health outcomes among human infants and toddlers. J Nutr. 2004;134(6):1432-1438. doi:10.1093/jn/134.6.1432 15173408

[6] Ettinger de Cuba S, Casey P, Cutts D, . Household food insecurity positively associated with increased hospital charges for infants. J Appl Res Child. 2018;9(1).

[7] Frank DA, Casey PH, Black MM, . Cumulative hardship and wellness of low-income, young children: multisite surveillance study. Pediatrics. 2010;125(5):e1115-e1123. doi:10.1542/peds.2009-1078 20385641

[8] Cook JT, Black M, Chilton M, . Are food insecurity’s health impacts underestimated in the U.S. population? marginal food security also predicts adverse health outcomes in young U.S. children and mothers. Adv Nutr. 2013;4(1):51-61. doi:10.3945/an.112.003228 23319123 PMC3648739

[9] Nsubuga P, White ME, Thacker SB, et al. Public health surveillance: a tool for targeting and monitoring interventions. In: Jamison DT, Breman JG, Measham AR, et al, eds; International Bank for Reconstruction and Development/The World Bank. *Disease Control Priorities in Developing Countries*. 2nd ed. Oxford University Press; 2006:chap 53. Accessed March 16, 2023. https://www.ncbi.nlm.nih.gov/books/NBK11770

[10] Connecting the brain to the rest of the body: early childhood development and lifelong health are deeply intertwined. National Scientific Council on the Developing Child, Center on the Developing Child at Harvard University. Working Paper No. 15. June 2020. Accessed February 26, 2023. https://developingchild.harvard.edu/resources/connecting-the-brain-to-the-rest-of-the-body-early-childhood-development-and-lifelong-health-are-deeply-intertwined/

[11] Tracking the COVID-19 economy’s effects on food, housing, and employment hardships. Center on Budget and Policy Priorities. 2022. Accessed February 26, 2023. https://www.cbpp.org/research/poverty-and-inequality/tracking-the-covid-19-economys-effects-on-food-housing-and

[12] Waxman E, Gupta P, Gonzalez D. Six months into the pandemic, 40 percent of parents with young children have experienced economic fallout. Urban Institute. December 8, 2020. Accessed February 26, 2023. https://www.urban.org/research/publication/six-months-pandemic-40-percent-parents-young-children-have-experienced-economic-fallout

[13] Maye A, Banerjee A, Johnson C. The dual crisis: how the COVID-19 recession deepens racial and economic inequality among communities of color. Center for Law and Social Policy. November 2020. Accessed February 26, 2023. https://www.clasp.org/sites/default/files/publications/2020/11/Jobs%20Brief%20Nov.%202020.pdf

[14] Zambrana RE, Williams DR. The intellectual roots of current knowledge on racism and health: relevance to policy and the national equity discourse. Health Aff (Millwood). 2022;41(2):163-170. doi:10.1377/hlthaff.2021.01439 35130075

[15] Williams DR, Lawrence JA, Davis BA. Racism and health: evidence and needed research. Annu Rev Public Health. 2019;40:105-125. doi:10.1146/annurev-publhealth-040218-043750 30601726 PMC6532402

[16] Robust COVID relief achieved historic gains against poverty and hardship, bolstered economy. Center on Budget and Policy Priorities. 2022. Accessed February 26, 2023. https://www.cbpp.org/research/poverty-and-inequality/robust-covid-relief-achieved-historic-gains-against-poverty-and

[17] Evich H. Food stamp spending jumped nearly 50 percent in 2020. *POLITICO*. Accessed February 26, 2023. https://www.politico.com/news/2021/01/27/food-stamp-spending-2020-463241

[18] More than 1.8 million additional economic impact payments disbursed under the American Rescue Plan; total payments reach nearly 167 million. US Department of Treasury. Accessed February 26, 2023. https://home.treasury.gov/news/press-releases/jy0201

[19] Saloner B, Campbell J, Gollust S, Blewett LA. Changes in material hardship during the first year of the COVID-19 pandemic. JAMA Health Forum. 2022;3(2):e215213. doi:10.1001/jamahealthforum.2021.5213 35977270 PMC8903113

[20] Perez-Lopez D, Bee C. Majority who received stimulus payments spending most of it on household expenses. Census Bureau. June 24, 2020. Accessed February 26, 2023. https://www.census.gov/library/stories/2020/06/how-are-americans-using-their-stimulus-payments.html

[21] Fix M, Gelatt J, Capps R. Nearly 3 million U.S. citizens and legal immigrants initially excluded under the CARES Act are covered under the December 2020 COVID-19 stimulus. Migration Policy Institute. January 2021. Accessed February 26, 2023. https://www.migrationpolicy.org/news/cares-act-excluded-citizens-immigrants-now-covered

[22] Haley J, Kenny G, Bernstein H, Gonzalez D. One in five adults in immigrant families with children report chilling effects in public benefit receipt in 2019. Urban Institute. June 18, 2020. Accessed February 26, 2023. https://www.urban.org/research/publication/one-five-adults-immigrant-families-children-reported-chilling-effects-public-benefit-receipt-2019

[23] Sommers BD, Allen H, Bhanja A, Blendon RJ, Orav EJ, Epstein AM. Assessment of perceptions of the public charge rule among low-income adults in Texas. JAMA Netw Open. 2020;3(7):e2010391-e2010391. doi:10.1001/jamanetworkopen.2020.10391 32667651 PMC7364340

[24] Nwadiuko J, German J, Chapla K, . Changes in health care use among undocumented patients, 2014-2018. JAMA Netw Open. 2021;4(3):e210763-e210763. doi:10.1001/jamanetworkopen.2021.0763 33666662 PMC7936260

[25] Patrick SW, Henkhaus LE, Zickafoose JS, . Well-being of parents and children during the COVID-19 pandemic: a national survey. Pediatrics. 2020;146(4):e2020016824. doi:10.1542/peds.2020-016824 32709738

[26] Children’s HealthWatch. Children’s HealthWatch: our study. Accessed March 14, 2023. https://childrenshealthwatch.org/our-survey

[27] Bovell-Ammon A, Ettinger de Cuba S, Lê-Scherban F, et al. Changes in economic hardships arising during the COVID-19 pandemic: differences by nativity and race. *J Immigr Minor Health*. 2023;25(2):483-488. doi:10.1007/s10903-022-01410-z PMC9638452 36334182

[28] Children’s HealthWatch. COVID-19 Follow-Up Study (CHW-COVID). 2023. Accessed March 14, 2023. https://childrenshealthwatch.org/covid-19-follow-up-study

[29] Blumberg SJ, Bialostosky K, Hamilton WL, Briefel RR. The effectiveness of a short form of the Household Food Security Scale. Am J Public Health. 1999;89(8):1231-1234. doi:10.2105/AJPH.89.8.1231 10432912 PMC1508674

[30] U.S. Household Food Security Survey Module: Six-Item Short Form. US Department of Agriculture Economic Research Service. 2012. Accessed March 14, 2023. https://www.ers.usda.gov/media/8282/short2012.pdf

[31] Children in U.S. immigrant families. Migration Policy Institute. Accessed February 26, 2023. https://www.migrationpolicy.org/programs/data-hub/charts/children-immigrant-families

[32] America’s children: key national indicators of well-being, 2021—demographic background. Federal Interagency Forum on Child and Family Statistics. 2021. Accessed March 14, 2023. https://www.childstats.gov/pdf/ac2021/ac_21.pdf

[33] Chilton M, Rose D. A rights-based approach to food insecurity in the United States. Am J Public Health. 2009;99(7):1203-1211. doi:10.2105/AJPH.2007.130229 19443834 PMC2696644

[34] Bernstein H, Gonzalez D, Karpman M. Adults in low-income immigrant families were deeply affected by the COVID-19 crisis yet avoided safety net programs in 2020. Urban Institute. May 2021. Accessed February 26, 2023. https://www.urban.org/sites/default/files/publication/104280/adults-in-low-income-immigrant-families-deeply-affected-by-pandemic-yet-avoided-safety-net_0.pdf

[35] Cooney P, Schaefer H. Material hardship and mental health following the COVID-19 relief bill and American Rescue Plan Act. *Poverty Solutions*. May 2021. Accessed February 26, 2023. https://sites.fordschool.umich.edu/poverty2021/files/2021/05/PovertySolutions-Hardship-After-COVID-19-Relief-Bill-PolicyBrief-r1.pdf

[36] Reimold AE, Grummon AH, Taillie LS, Brewer NT, Rimm EB, Hall MG. Barriers and facilitators to achieving food security during the COVID-19 pandemic. Prev Med Rep. 2021;23:101500. doi:10.1016/j.pmedr.2021.101500 34401218 PMC8348548

[37] Marr C, Jacoby S, Huang C, Hingtgen S, Sherman A, Beltran J. Future stimulus should include immigrants and dependents previously left out, mandate automatic payments. Center on Budget and Policy Priorities. May 6, 2020. Accessed February 26, 2023. https://www.cbpp.org/research/economy/future-stimulus-should-include-immigrants-and-dependents-previously-left-out

[38] Burnside A. 10 things to know about the second round of stimulus payments. Center for Law and Social Policy. Accessed February 26, 2023. January 4, 2021. https://www.clasp.org/blog/10-things-know-about-second-round-stimulus-payments/

[39] Marr C, Cox K, Bryant K, Dean S, Caines R, Sherman A. Aggressive state outreach can help reach the 12 million non-filers eligible for stimulus payments. Center on Budget and Policy Priorities. Updated October 14, 2020. Accessed February 26, 2023. https://www.cbpp.org/research/federal-tax/aggressive-state-outreach-can-help-reach-the-12-million-non-filers-eligible

[40] Bovell-Ammon A, Cuba SE, Coleman S, . Trends in food insecurity and SNAP participation among immigrant families U.S.-born young children. Children (Basel). 2019;6(4):55. doi:10.3390/children6040055 30987395 PMC6517901

[41] Kinsey EW, Kinsey D, Rundle AG. COVID-19 and food insecurity: an uneven patchwork of responses. *J Urban Health*. 2020;97(3):332–335. doi:10.1007/s11524-020-00455-5 PMC7274516 32504251

[42] Barofsky J, Vargas A, Rodriguez D, Barrows A. Spreading fear: the announcement of the public charge rule reduced enrollment in child safety-net programs. Health Aff (Millwood). 2020;39(10):1752-1761. doi:10.1377/hlthaff.2020.00763 33017237

[43] Callaghan T, Washburn DJ, Nimmons K, Duchicela D, Gurram A, Burdine J. Immigrant health access in Texas: policy, rhetoric, and fear in the Trump era. BMC Health Serv Res. 2019;19(1):342. doi:10.1186/s12913-019-4167-1 31164114 PMC6549327

[44] Pino LJ. Immigration policy and perception impacts on SNAP access and eligibility: a view from the field. *Renewable Agriculture and Food Systems*. 2020;35(4):416-419. doi:10.1017/S1742170519000449

[45] Bernstein H, Karpman M, Gonzalez D, Zuckerman S. Immigrant families continued avoiding the safety net during the COVID-19 crisis. Urban Institute. February 2021. Accessed February 26, 2023. https://www.urban.org/sites/default/files/publication/103565/immigrant-families-continued-avoiding-the-safety-net-during-the-covid-19-crisis.pdf

[46] Shafer PR, Gutiérrez KM, Ettinger de Cuba S, Bovell-Ammon A, Raifman J. Association of the implementation of child tax credit advance payments with food insufficiency in US households. JAMA Netw Open. 2022;5(1):e2143296-e2143296. doi:10.1001/jamanetworkopen.2021.43296 35024837 PMC8759005

[47] Parolin Z, Ananat E, Collyer S, Curran M, Wimer C. The initial effects of the expanded child tax credit on material hardship. National Bureau of Economic Research working paper 29285. 2021. Accessed February 26, 2023. https://www.nber.org/papers/w29285

[48] Bovell-Ammon A, Burnett D, Ettinger de Cuba S, . ʻI didn’t have to worryʼ—how the child tax credit helped families catch up on rent and improved health. Children’s HealthWatch; Revolutionary Healing; Kairos Center for Religions, Rights, and Social Justice. August 2022. Accessed February 26, 2023. https://childrenshealthwatch.org/wp-content/uploads/CTC-Report-Aug-2022-Final.pdf

[49] Katz VS. What it means to be “under-connected” in lower-income families. J Child Media. 2017;11(2):241-244. doi:10.1080/17482798.2017.1305602

[50] Child and Adolescent Health Measurement Initiative. The National Survey of Children’s Health. Data Resource Center for Child and Adolescent Health. Accessed March 11, 2023. https://www.childhealthdata.org/learn-about-the-nsch/NSCH

[51] Bovell-Ammon A, Mansilla C, Poblacion A, . Housing intervention for medically complex families associated with improved family health: pilot randomized trial. Health Aff (Millwood). 2020;39(4):613-621. doi:10.1377/hlthaff.2019.01569 32250672

