# Supplementary material for: Association Between Material Hardship in Families With Young Children and Federal Relief Program Participation by Race and Ethnicity and Maternal Nativity
Source: JAMA Health Forum. 2023 Apr 21;4(4):e230508. doi: 10.1001/jamahealthforum.2023.0508 (PMC10122179; doi:10.1001/jamahealthforum.2023.0508)
Supplement: Supplement 1. — Data Sharing Statement [file jamahealthforum-e230508-s001.pdf]

Data Sharing Statement

Lê-Scherban. Association Between Material Hardship in Families With Young Children and Federal Relief Program Participation by Race and Ethnicity and Maternal Nativity. *JAMA Health Forum*. Published April 21, 2023. doi:10.1001/jamahealthforum.2023.0508

Data
Data available: No
